# Multicenter Evaluation of the ePlex Respiratory Pathogen Panel for the Detection of Viral and Bacterial Respiratory Tract Pathogens in Nasopharyngeal Swabs

**DOI:** 10.1128/JCM.01658-17

**Published:** 2018-01-24

**Authors:** N. Esther Babady, Matthew R. England, Kristen L. Jurcic Smith, Taojun He, Dona Saumya Wijetunge, Yi-Wei Tang, Robin R. Chamberland, Marilyn Menegus, Ella M. Swierkosz, Robert C. Jerris, Wallace Greene

**Affiliations:** a Memorial Sloan Kettering Cancer Center, New York, New York, USA; b Pennsylvania State University, Hershey Medical Center, Hershey, Pennsylvania, USA; c University of Rochester Medical Center, Rochester, New York, USA; d The Eighth Affiliated Hospital of Sun Yat-sen University, Shenzhen, China; e Weill Medical College of Cornell University, New York, New York, USA; f Saint Louis University School of Medicine, St. Louis, Missouri, USA; g Children's Healthcare of Atlanta, Atlanta, Georgia, USA; Boston Children's Hospital

**Keywords:** respiratory tract infections, multiplex syndromic panel, rapid diagnosis, sample-to-answer test, rapid PCR, respiratory pathogens

## Abstract

The performance of the new ePlex Respiratory Pathogen (RP) panel (GenMark Diagnostics) for the simultaneous detection of 19 viruses (influenza A virus; influenza A H1 virus; influenza A 2009 H1 virus; influenza A H3 virus; influenza B virus; adenovirus; coronaviruses [HKU1, OC43, NL63, and 229E]; human rhinovirus/enterovirus; human metapneumovirus; parainfluenza viruses 1, 2, 3, and 4; and respiratory syncytial virus [RSV] [RSV subtype A and RSV subtype B]) and 2 bacteria (Mycoplasma pneumoniae and Chlamydia pneumoniae) was evaluated. Prospectively and retrospectively collected nasopharyngeal swab (NPS) specimens (*n* = 2,908) were evaluated by using the ePlex RP panel, with the bioMérieux/BioFire FilmArray Respiratory Panel (BioFire RP) as the comparator method. Discordance analysis was performed by using target-specific PCRs and bidirectional sequencing. The reproducibility of the assay was evaluated by using reproducibility panels comprised of 6 pathogens. The overall agreement between the ePlex RP and BioFire RP results was >95% for all targets. Positive percent agreement with the BioFire RP result for viruses ranged from 85.1% (95% confidence interval [CI], 80.2% to 88.9%) to 95.1% (95% CI, 89.0% to 97.9%), while negative percent agreement values ranged from 99.5% (95% CI, 99.1% to 99.7%) to 99.8% (95% CI, 99.5% to 99.9%). Additional testing of discordant targets (12%; 349/2,908) confirmed the results of ePlex RP for 38% (131/349) of samples tested. Reproducibility was 100% for all targets tested, with the exception of adenovirus, for which reproducibilities were 91.6% at low virus concentrations and 100% at moderate virus concentrations. The ePlex RP panel offers a new, rapid, and sensitive “sample-to-answer” multiplex panel for the detection of the most common viral and bacterial respiratory pathogens.

## INTRODUCTION

Respiratory tract infections are a significant contributor to the global burden of respiratory tract illnesses, with up to 4 million deaths worldwide in 2013 (1). Several pathogens, including viruses and bacteria, can cause respiratory tract infections. As clinical presentations often overlap, identification of the underlying pathogen based solely on clinical criteria is challenging (2). Even though most respiratory tract infections are self-limited in immunocompetent hosts, many are treated, as shown in a recent study highlighting the increase in unnecessary antibiotic use in this group (3). In other patient groups, including immunocompromised patients, elderly patients, and critically ill patients, complications from respiratory tract infections often occur, with poor outcomes including increased morbidity and mortality (4–6). Thus, the ability to rapidly and accurately diagnose respiratory tract infections is critical for optimal patient care. Additionally, rapid and accurate diagnosis is essential for the timely administration of antiviral therapy, when available, and the institution of contact and droplet precautions to prevent health care-associated transmission (7, 8). Several instruments and multiplexed molecular panels are currently approved by the U.S. Food and Drug Administration (FDA) for the rapid and sensitive detection of viral and bacterial respiratory pathogens in nasopharyngeal swab (NPS) specimens. These methods differ based on several criteria, including the degree of multiplexing (4 to 22 targets), complexity of the method (moderate to high), throughput (low to high), and turnaround time (TAT) (∼1 h to 8 h) (9). In general, performance characteristics of these multiplex panels are significantly better than those of conventional methods, including viral culture and antigen tests, and have replaced these methods in many laboratories. On the other hand, the increased cost of multiplexed molecular panels compared to the cost of conventional methods has raised questions on their cost/benefit ratio, particularly as many viral targets on these panels have no specific treatments. However, given their utility in the rapid diagnosis of infections (especially in immunocompromised patients), infection control and prevention practices, and antibiotic stewardship, the interest in multiplexed PCRs remains high.

The GenMark Respiratory Pathogen (RP) panel is a qualitative nucleic acid multiplex test that recently received U.S. FDA clearance for use on GenMark's new ePlex instrument. The ePlex RP panel detects 21 of the most common respiratory pathogens in NPS specimens. Viral targets identified are adenovirus, coronavirus (229E, HKU1, NL63, and OC43), human metapneumovirus (hMPV), human rhinovirus/enterovirus (HRV/EV), influenza A virus, influenza A H1 virus, influenza A 2009 H1 virus, influenza A H3 virus, influenza B virus, parainfluenza virus 1 (PIV1), PIV2, PIV3, PIV4, respiratory syncytial virus subtype A (RSV-A), and RSV-B. Bacterial targets identified are Chlamydia pneumoniae and Mycoplasma pneumoniae (10). The ePlex RP panel is a sample-to-answer multiplex assay that runs on a single-use cartridge that automates all aspects of nucleic acid testing, including extraction, amplification, and detection. It combines electrowetting microfluidics and GenMark's eSensor technology, which is based on the principles of competitive DNA hybridization and electrochemical detection, as previously described for the GenMark XT-8 Respiratory Viral panel (11).

In this multicenter clinical trial study, the performance characteristics of the investigational-use-only (IUO) ePlex RP panel were evaluated by using NPS specimens collected at 13 sites across the United States and Canada. Reproducibility was evaluated at 3 sites. In addition to assay performance, other characteristics, including assay workflow and turnaround time, were evaluated.

## MATERIALS AND METHODS

### Study population.

The study population included patients of all ages and both genders presenting with signs and/or symptoms of upper respiratory tract infection at 13 clinical sites located in the United States and Canada. Residual NPS specimens were prospectively collected from March 2013 through August 2014 from 5 sites and frozen at −80°C for future testing and were prospectively collected from September through October 2016 from 4 sites and tested fresh. To supplement the results of the prospective collection, NPS samples positive for low-prevalence pathogens were retrospectively collected and frozen. Frozen samples were thawed, pipetted into separate aliquots, and frozen at −80°C until they were tested by ePlex RP, the BioFire RP, and/or target-specific PCR/bidirectional sequencing as needed. Also, for low-prevalence pathogens, additional contrived samples were used to evaluate assay performance.

### Overall study design.

NPS specimens were prospectively and retrospectively collected in viral transport medium, handled, and processed according to the NPS kit manufacturer's instructions. Either samples were collected specifically for this study, separately from a standard-of-care (SOC) purpose, or the residual sample was deidentified and provided after SOC was completed. The study was approved by a central quorum review institutional review board and/or individual clinical testing site institutional review board.

Samples were tested with the ePlex RP panel at 1 of 5 sites and compared to results of testing with BioFire FilmArray respiratory panel version 1.7 (bioMérieux, Durham, NC) and an analytically validated PCR amplification assay(s) followed by bidirectional sequencing, if necessary (e.g., for resolution of discordant results or virus subtyping) (Laboratory Corporation of America, Morrisville, NC).

### GenMark ePlex RP panel testing.

**FIG 1:**
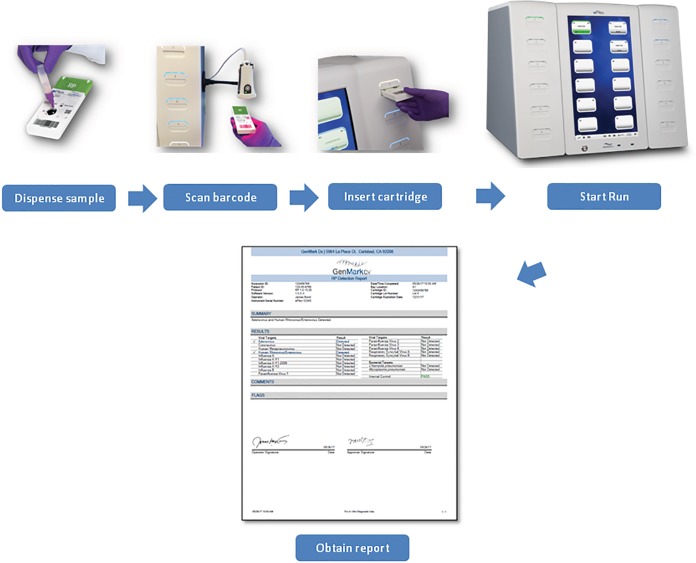
ePlex respiratory panel assay workflow.

Testing with the ePlex RP panel was performed according to the manufacturer's instructions, using the materials in the kit. Briefly, after vortexing, 200 μl of the primary NPS sample was aspirated into the sample delivery device (SDD) provided with the ePlex RP panel kit and vortexed. The entire volume of the SDD was dispensed into the sample loading port of the ePlex RP panel cartridge, and the cap was depressed to close the port. Each cartridge was bar-coded and scanned with the ePlex instrument and inserted into an available bay (Fig. 1). Upon the completion of the assay run, the ePlex instrument ejected the cartridge for disposal, and an ePlex RP panel report was generated.

### Comparator method and method for resolution of discordant results.

The comparator method was BioFire FilmArray respiratory panel version 1.7. All collected samples were tested with the BioFire RP, which detects all the respiratory viral and bacterial targets included on the ePlex RP panel but does not differentiate between RSV subtypes A and B (12). Samples with RSV detected by the BioFire RP were additionally tested with an analytically validated PCR amplification assay(s) followed by confirmation by bidirectional sequencing to determine the subtype (Laboratory Corporation of America, Morrisville, NC).

Results from collected samples that were discordant between the ePlex RP panel and the comparator method (i.e., false negative [FN] or false positive [FP]) were tested with an analytically validated PCR amplification assay(s) followed by bidirectional sequencing as described above. For coronaviruses, additional repeat testing by the BioFire RP and ePlex RP panel was also conducted as part of discordant-result resolution.

### Reproducibility study.

Three reproducibility panels, each with 6 pathogens (influenza A H3 virus, RSV-A, PIV1, hMPV, coronavirus OC43, and adenovirus species B) at 3 different concentrations (moderate [3× the limit of detection {LOD}]), low [1× LOD], and negative) in NPS specimens, were tested at 3 sites. LOD studies were performed by the manufacturer (data not shown). Reproducibility testing was performed by 2 operators at each of 3 sites, and each operator tested the reproducibility panels in triplicate over 6 days (including 5 nonconsecutive days) by using 3 different cartridge lots, resulting in 2 days of testing for each of the 3 lots.

### Statistical methods.

Positive percent agreement (PPA), negative percent agreement (NPA), and overall percent agreement (OPA) with the comparator method results or expected results were determined for each target detected by the ePlex RP panel. The PPA was calculated as 100 × no. of TP/(no. of TP + no. of FN), the NPA was calculated as 100 × no. of TN/(no. of TN + no. of FP), and OPA was calculated as 100 × (no. of TP + no. of TN)/(no. of TP + no. of TN + no. of FP + no. of FN), where TP is true-positive results, FN is false-negative results, TN is true-negative results, and FP is false-positive results. The two-sided 95% score confidence interval (CI) was calculated for PPA, NPA, and OPA. Statistical analysis was performed by using SAS version 9.4.

### Workflow and turnaround time study.

A time study was performed to determine the time needed to perform the ePlex RP panel test. Data were averaged from 20 samples tested by 2 operators. Each step of the process from sample preparation to unloading of the instrument was recorded and timed, and an average TAT was determined.

## RESULTS

### Patient demographics.

A total of 2,462 prospectively collected, 446 retrospectively collected, and 327 contrived samples were eligible for testing with the ePlex RP panel. Approximately 67% of samples were collected with Copan-manufactured UTM (universal transport medium), and 33% were collected with Remel M4 or M5 medium. Demographic information for the evaluable prospectively and retrospectively collected subject samples is provided in Table 1. Approximately 50% of the subjects were male, with median ages of 33 years for subjects with prospectively collected samples and 5 years for subjects with retrospectively collected samples. The prevalences of ePlex RP panel targets by age group during the two phases of prospective collection are provided in Tables S1 and S2 in the supplemental material.

**TABLE 1:** Subject demographics by collection type

Parameter	Value for sample type	
	Prospective (*n* = 2,462)	Retrospective (*n* = 446)
No. (%) of subjects of gender		
Male	1,247 (50.6)	232 (52.0)
Female	1,215 (49.4)	214 (48.0)
Age (yr)		
Mean (SD)	35.2 (29.6)	23.5 (29.0)
Median (range)	33.0 (0.1–101.0)	5.0 (0.1–95.0)
No. (%) of subjects in age group		
<1 yr	388 (15.8)	122 (27.4)
1–5 yr	325 (13.2)	107 (24.0)
5–21 yr	321 (13.0)	59 (13.2)
21–65 yr	926 (37.6)	99 (22.2)
>65 yr	502 (20.4)	59 (13.2)

### Assay performance. (i) Accuracy.

Of the 3,235 samples eligible for testing with the ePlex RP panel, 154 had invalid results (4.8%). After repeat testing, 8 samples were not evaluable due to final, invalid results (7 prospective samples and 1 contrived sample). The final validity rate was 99.8% (95% CI, 99.5% to 99.9%). The ePlex RP panel detected a potential respiratory pathogen in 1,212 of 2,462 prospectively collected NPS specimens tested, for a positivity rate of 49%. The PPAs and NPAs with 95% CIs of the ePlex RP panel targets with comparator methods are provided in Tables 2 to 4 separately for retrospectively collected samples and prospectively collected samples and with all samples combined. Additional data for fresh and frozen prospectively collected samples are presented in Table S3 in the supplemental material.

**TABLE 2:** Results for retrospective samples^*a*^

Target	No. of TP/no. of TP + FN	PPA (%) (95% CI)	No. of TN/no. of TN + FP	NPA (%) (95% CI)
Adenovirus	55/56	98.2 (90.6–99.7)	386/390	99.0 (97.4–99.6)
Coronavirus	121/138	87.7 (81.2–92.2)	307/307	100 (98.8–100)
Human metapneumovirus	5/7	71.4 (35.9–91.8)	439/439	100 (99.1–100)
Human rhinovirus/enterovirus	37/41	90.2 (77.5–96.1)	384/402	95.5 (93.0–97.1)
Influenza A virus	75/82	91.5 (83.4–95.8)	363/363	100 (99.0–100)
Influenza A H1 virus	0/0		446/446	100 (99.1–100)
Influenza A 2009 H1N1 virus	27/31	87.1 (71.1–94.9)	415/415	100 (99.1–100)
Influenza A H3 virus	45/51	88.2 (76.6–94.5)	394/394	100 (99.0–100)
Influenza B virus	1/1	100 (20.7–100)	445/445	100 (99.1–100)
Parainfluenza virus 1	43/48	89.6 (77.8–95.5)	396/397	99.7 (98.6–100)
Parainfluenza virus 2	46/51	90.2 (79.0–95.7)	395/395	100 (99.0–100)
Parainfluenza virus 3	2/2	100 (34.2–100)	444/444	100 (99.1–100)
Parainfluenza virus 4	18/20	90.0 (69.9–97.2)	426/426	100 (99.1–100)
Respiratory syncytial virus subtype A	25/27	92.6 (76.6–97.9)	414/414	100 (99.1–100)
Respiratory syncytial virus subtype B	21/22	95.5 (78.2–99.2)	419/419	100 (99.1–100)
Chlamydia pneumoniae	1/1	100 (20.7–100)	445/445	100 (99.1–100)
Mycoplasma pneumoniae	7/7	100 (64.6–100)	439/439	100 (99.1–100)

a FP, false-positive results; FN, false-negative results; TP, true-positive results; TN, true-negative results.

**TABLE 3:** Results for prospective samples^*a*^

Target	No. of TP/no. of TP + FN	PPA (%) (95% CI)	No. of TN/no. of TN + FP	NPA (%) (95% CI)
Adenovirus	54/61	88.5 (78.2–94.3)	2,373/2,401	98.8 (98.3–99.2)
Coronavirus	96/117	82.1 (74.1–88.0)	2,331/2,345	99.4 (99.0–99.6)
Human metapneumovirus	107/113	94.7 (88.9–97.5)	2,343/2,349	99.7 (99.4–99.9)
Human rhinovirus/enterovirus	493/519	95.0 (92.8–96.6)	1,860/1,943	95.7 (94.7–96.5)
Influenza A virus	106/111	95.5 (89.9–98.1)	2,347/2,351	99.8 (99.6–99.9)
Influenza A H1 virus	0/0		2,462/2,462	100 (99.8–100)
Influenza A 2009 H1N1 virus	70/71	98.6 (92.4–99.8)	2,385/2,391	99.7 (99.5–99.9)
Influenza A H3 virus	34/37	91.9 (78.7–97.2)	2,425/2,425	100 (99.8–100)
Influenza B virus	59/66	89.4 (79.7–94.8)	2,391/2,396	99.8 (99.5–99.9)
Parainfluenza virus 1	24/25	96.0 (80.5–99.3)	2,436/2,437	100 (99.8–100)
Parainfluenza virus 2	21/22	95.5 (78.2–99.2)	2,438/2,440	99.9 (99.7–100)
Parainfluenza virus 3	99/109	90.8 (83.9–94.9)	2,348/2,353	99.8 (99.5–99.9)
Parainfluenza virus 4	8/8	100 (67.6–100)	2,447/2,454	99.7 (99.4–99.9)
Respiratory syncytial virus subtype A	35/40	87.5 (73.9–94.5)	2,418/2,419	100 (99.8–100)
Respiratory syncytial virus subtype B	90/96	93.8 (87.0–97.1)	2,361/2,363	99.9 (99.7–100)
Chlamydia pneumoniae	2/5	40.0 (11.8–76.9)	2,456/2,457	100 (99.8–100)
Mycoplasma pneumoniae	7/8	87.5 (52.9–97.8)	2,452/2,454	99.9 (99.7–100)

a FP, false-positive results; FN, false-negative results; TP, true-positive results; TN, true-negative results; PPA, positive percent agreement; NPA, negative percent agreement (defined in comparison to the comparator method); CI, confidence interval.

**TABLE 4:** Results for all clinical prospective and retrospective samples^*a*^

Target	No. of TP/no. of TP + FN	PPA (%) (95% CI)	No. of TN/no. of TN + FP	NPA (%) (95% CI)	OPA (%) (95% CI)
Adenovirus	109/117	93.2 (87.1–96.5)	2,759/2,791	98.9 (98.4–99.2)	98.6 (98.1–99.0)
Coronavirus	217/255	85.1 (80.2–88.9)	2,638/2,652	99.5 (99.1–99.7)	98.2 (97.7–98.6)
Human metapneumovirus	112/120	93.3 (87.4–96.6)	2,782/2,788	99.8 (99.5–99.9)	99.5 (99.2–99.7)
Human rhinovirus/enterovirus	530/560	94.6 (92.5–96.2)	2,244/2,345	95.7 (94.8–96.4)	95.5 (94.7–96.2)
Influenza A virus^*b*^	181/193	93.8 (89.4–96.4)	2,710/2,714	99.9 (99.6–99.9)	99.4 (99.1–99.7)
Influenza A H1 virus	0/0		2,908/2,908	100 (99.9–100)	100 (99.9–100)
Influenza A 2009 H1N1 virus	97/102	95.1 (89.0–97.9)	2,800/2,806	99.8 (99.5–99.9)	99.6 (99.3–99.8)
Influenza A H3 virus	79/88	89.8 (81.7–94.5)	2,819/2,819	100 (99.9–100)	99.7 (99.4–99.8)
Influenza B virus	60/67	89.6 (80.0–94.8)	2,836/2,841	99.8 (99.6–99.9)	99.6 (99.3–99.8)
Parainfluenza virus 1	67/73	91.8 (83.2–96.2)	2,832/2,834	99.9 (99.7–100)	99.7 (99.5–99.9)
Parainfluenza virus 2	67/73	91.8 (83.2–96.2)	2,833/2,835	99.9 (99.7–100)	99.7 (99.5–99.9)
Parainfluenza virus 3	101/111	91.0 (84.2–95.0)	2,792/2,797	99.8 (99.6–99.9)	99.5 (99.2–99.7)
Parainfluenza virus 4	26/28	92.9 (77.4–98.0)	2,873/2,880	99.8 (99.5–99.9)	99.7 (99.4–99.8)
Respiratory syncytial virus subtype A	60/67	89.6 (80.0–94.8)	2,832/2,833	100 (99.8–100)	99.7 (99.5–99.9)
Respiratory syncytial virus subtype B	111/118	94.1 (88.3–97.1)	2,780/2,782	99.9 (99.7–100)	99.7 (99.4–99.8)
Chlamydia pneumoniae	3/6	50.0 (18.8–81.2)	2,901/2,902	100 (99.8–100)	99.9 (99.6–99.9)
Mycoplasma pneumoniae	14/15	93.3 (70.2–98.8)	2,891/2,893	99.9 (99.7–100)	99.9 (99.7–100)

a FP, false-positive result; FN, false-negative result; TP, true-positive result; TN, true-negative result. PPA, positive percent agreement; NPA, negative percent agreement; OPA, overall percent agreement (defined in comparison to the comparator method); CI, confidence interval.

b Influenza A virus comparator results detected 102 samples with A 2009 H1, 88 with A H3, and 3 with no subtype.

The overall percent agreement between the ePlex RP and BioFire RP results was >95% for all targets tested. The PPA ranged from 85.1% (95% CI, 80.2% to 88.9%) for coronaviruses to 95.1% (95% CI, 89.0% to 97.9%) for influenza A 2009 H1N1 virus, with NPAs of 99.5% (95% CI, 99.1% to 99.7%) and 99.8% (95% CI, 99.5% to 99.9%), respectively (Table 4). For 6 samples, ePlex RP was negative for the influenza A virus target but positive for either the 2009 H1 (*n* = 5) or H3 (*n* = 1) subtype, and 9 samples were positive for the influenza A virus target without a subtype being identified. Of note, influenza A H1 virus was not detected, and very few Chlamydia pneumoniae (*n* = 4) isolates were detected during the study period. For these targets, at least 50 specimens with the pathogen spiked into negative NPS specimens were contrived; all were detected by ePlex RP (data not shown).

Discordance resolution results (Table 5) demonstrate that while ePlex RP had a high level of agreement with the BioFire RP and detected the same targets in nearly all samples, each assay detected targets that the other did not. In Table 5, samples with targets that were detected by ePlex RP but not detected by BioFire RP (FP) are counted as true-positive samples if the target was detected by discordance testing (TPd). Samples with targets not detected by ePlex RP but detected by the BioFire RP (FN) are counted as true-negative samples if the target was not detected by discordance testing (TNd). Given the number of discordant samples between the two panels for coronavirus detection, additional repeat testing by both the BioFire RP and ePlex RP was performed as part of the discordance analysis. This additional testing demonstrated evidence of decreased sample stability over time or variability in aliquots tested. Thus, for coronaviruses, a FN sample was counted as a true-positive sample if the target was detected by repeat testing by ePlex RP (TPd). A total of 26 samples were not tested by PCR assays for a variety of reasons, including an insufficient volume for repeat testing, which included 8 samples with FN results (1 for coronavirus, 1 for hMPV, 4 for HRV/EV, and 2 for influenza A virus) and 18 samples with FP HRV/EV results.

**TABLE 5:** Discordance testing and resolution results

Target	Total no. of samples	No. of ePlex^+^/BioFire^+^ results (TP)	No. of ePlex^+^/BioFire^−^ results (FP)	No. of ePlex^−^/BioFire^+^ results (FN)	No. of ePlex^−^/BioFire^−^ results (TN)	No. of samples with discordance resolution result^*a*^			
						FP		FN	
						TPd	FPd	TNd	FNd
Adenovirus	2,908	109	32	8	2,759	15	17	4	4
Coronavirus	2,907	217	14	38^*b*^	2,638	11^*b*^	11	12	18^*c*^
Human metapneumovirus	2,908	112	6	8	2,782	4	2	1	7^*c*^
Human rhinovirus/enterovirus	2,905	530	101	30	2,244	42	59^*c*^	7	23^*c*^
Influenza A virus	2,907	181	4	12	2,710	1	3	4	8^*c*^
Influenza A H1 virus	2,908	0	0	0	2,908	0	0	0	0
Influenza A 2009 H1N1 virus	2,908	97	6	5	2,800	4	2	2	3
Influenza A H3 virus	2,907	79	0	9	2,819	0	0	2	7
Influenza B virus	2,908	60	5	7	2,836	2	3	3	4
Parainfluenza virus 1	2,907	67	2	6	2,832	0	2	2	4
Parainfluenza virus 2	2,908	67	2	6	2,833	0	2	0	6
Parainfluenza virus 3	2,908	101	5	10	2,792	4	1	3	7
Parainfluenza virus 4	2,908	26	7	2	2,873	3	4	0	2
Respiratory syncytial virus subtype A	2,900	60	1	7	2,832	0	1	0	7
Respiratory syncytial virus subtype B	2,900	111	2	7	2,780	1	1	0	7
Chlamydia pneumoniae	2,908	3	1	3	2,901	1	0	1	2
Mycoplasma pneumoniae	2,908	14	2	1	2,891	1	1	1	0

a ePlex-positive (ePlex^+^)/BioFire-negative (BioFire^−^) (false-positive [FP]) results are counted as TPd if detected by discordance resolution (true positive [TP] after discordance testing resolution). ePlex^−^/BioFire^+^ (false-negative [FN]) results are counted as TNd if not detected by discordance resolution (true negative [TN] after discordance testing resolution) or as TPd if detected by repeat ePlex RP testing. Otherwise, discordance resolution results remain as false-positive or false-negative results (i.e., FP is FPd, and FN is FNd).

b Twenty samples with FN coronavirus results were repeat tested with ePlex RP as part of the discordance analysis. Eight of 20 samples had coronavirus detected upon repeat testing and were counted as 8 TPd samples, and 3 were confirmed by discordance testing.

c One FN coronavirus, 1 FN human metapneumovirus, 18 FP and 4 FN human rhinovirus/enterovirus, and 2 FN influenza A virus samples did not have discordance testing done, so their discordant FP, FN, TN, and TP results are defined in comparison to the comparator method.

Of the 2,462 prospectively collected samples, 164 had codetections by at least one of the assays (data not shown). The ePlex RP panel identified a total of 135 prospective samples with multiple pathogens detected, or 5.5% of all prospectively collected samples, while the BioFire RP detected a total of 116 samples with multiple pathogens, or 4.7% of all prospectively collected samples. Of the 135 samples with multiple pathogens detected by ePlex RP, 118 (4.8%) had two pathogens, 14 (0.6%) had three pathogens, and 3 (0.1%) had four pathogens detected. Of the 135 samples with codetections, 57 samples (2.3%) included one or more pathogens that were not detected by the BioFire RP method. The comparator method identified a total of 32 (1.3%) codetections with one or more pathogens not identified by the ePlex RP panel. Discordance resolution results are included in the analysis presented in Table 5.

### (ii) Reproducibility.

Each of 3 sites performing the reproducibility study collected 35 to 36 results for each target at each concentration, for a total of 107 to 108 samples per site. For the moderately positive and the negative panels, the percent agreement was 100% (95% CI, 96.6% to 100%) for all 7 panel targets. For the low-positive panel, the percent agreement was 100% (95% CI, 96.5% to 100%) for 6 of the 7 panel targets. For the remaining adenovirus panel target, the percent agreement in the low-positive panel was 91.6% (95% CI, 84.8% to 95.5%). Agreement results are shown in Table 6. A total of 315/324 (97.2%) samples yielded a valid result upon initial testing. Upon repeat testing, all but one specimen yielded a valid result, for a final validity rate of 99.7% (95% CI: 98.3% to 99.9%). No significant sources of variability were found for any of the targets on the panel across cartridge lots, operation days, technologists/operators, or sites (data not shown).

**TABLE 6:** Reproducibility data

Pathogen	LOD (TCID_50_/ml)^*a*^	Moderately positive results^*b*^			Low-positive results^*c*^			Negative results		
		Agreement^*d*^	% agreement	95% CI	Agreement^*d*^	% agreement	95% CI	Agreement^*d*^	% agreement	95% CI
Influenza A H3 virus	1 × 10^1^	108/108	100	96.6–100	107/107	100	96.5–100	108/108	100	96.6–100
Respiratory syncytial virus subtype A	1.5 × 10^0^	108/108	100	96.6–100	107/107	100	96.5–100	108/108	100	96.6–100
Adenovirus species B	2 × 10^0^	108/108	100	96.6–100	98/107	91.6	84.8–95.5	108/108	100	96.6–100
Parainfluenza virus 1	4 × 10^−1^	108/108	100	96.6–100	107/107	100	96.5–100	108/108	100	96.6–100
Human metapneumovirus	2 × 10^−1^	108/108	100	96.6–100	107/107	100	96.5–100	108/108	100	96.6–100
Coronavirus OC43	5 × 10^2^	108/108	100	96.6–100	107/107	100	96.5–100	108/108	100	96.6–100
Influenza A virus	3 × 10^−1^	108/108	100	96.6–100	107/107	100	96.5–100	108/108	100	96.6–100

a LOD, limit of detection; TCID_50_, 50% tissue culture infective dose.

b Moderately positive is defined as 3× LOD.

c Low positive is defined as 1× LOD.

d No. of observed results/no. of expected results.

### Workflow and turnaround time study.

The time to identification from receipt to result was determined to be 1 h 45 min and 57 s, with hands-on times (HOTs) per sample of 1 min and 35 s.

## DISCUSSION

In this multicenter clinical study, the performance characteristics of the new ePlex Respiratory Pathogen panel on the GenMark ePlex instrument were estimated by measuring agreement with the results of the BioFire RP, a commercially available multiplex respiratory panel. The ePlex RP panel was recently FDA cleared and expands the available options for laboratories interested in the rapid diagnosis of respiratory tract infections using broadly multiplexed molecular assay on NPS specimens. Similar to the BioFire RP, ePlex RP offers a large panel of both viral and bacterial respiratory pathogens in a simple, sample-to-answer format with minimal hands-on time and a rapid turnaround time to results.

As with all molecular diagnostic test methods, both the BioFire RP and ePlex RP have the potential to generate false-positive and false-negative results, as confirmed in this study by additional testing with target-specific PCRs with bidirectional sequencing. The unavailability of an established gold standard prevents a true measure of correctness, requiring the use of agreement measures with a nonreference standard instead to establish the performance characteristics of a new test (13). Thus, data presented in this study reflect only the agreement between the results of the two platforms. In spite of these limitations, the overall performance of ePlex RP was comparable to that of the BioFire RP, with an overall percent agreement (true-positive and true-negative results) of >95% for all available targets tested. In line with our results, a recent study by Nijhuis and colleagues evaluating the performance of the ePlex research-use-only (RUO) RP panel compared to laboratory-developed assays found an overall agreement of 97.4%, with discordant results occurring primarily for samples with low pathogen loads, as estimated by using cycle threshold (*C_T_*) values (i.e., high *C_T_* value, >35) (10).

The highest numbers of discordant results were observed for adenovirus, coronavirus, and rhinovirus/enterovirus targets. This finding may be explained by the large diversity of genotypes present within each of these groups of pathogens or the prevalence of these pathogens in the population tested. For adenovirus, the performance of diagnostic tests is particularly important for young children and immunocompromised hosts. However, the detection of adenovirus can be challenging. In this study, ePlex RP detected more adenoviruses than did the BioFire RP. A recent report on the performance of BioFire RP v1.7 for the detection of adenovirus highlighted the decreased sensitivity of the assay for samples with low viral loads or those positive for species A, D, and F (14).

Although the ePlex RP panel detects 4 genotypes of coronaviruses (HKU1, OC43, 229E, and NL63), results are reported without the genotyping information. There are currently no treatments for coronaviruses, and the utility of the genotyping information may lie in its value for epidemiological studies. Reports of severe coronavirus infections have been reported for immunocompromised hosts, but there has not been any indication that severity is associated with a particular genotype (15).

Similar to the BioFire RP, ePlex RP detects a wide range of rhinovirus and enteroviruses genotypes, but given their high degree of genetic similarity, it does not reliably differentiate between these two species. Based on discordance analysis performed by PCR and bidirectional sequencing, most samples were positive for rhinovirus, a trend that is expected given that rhinoviruses are the most common cause of the common cold (16).

The overall agreement for influenza viruses was high. As the prospective collection periods for this clinical study occurred during the 2013 and 2016 respiratory seasons, there was an expected lack of clinical specimens positive for influenza A H1 virus (17). Most of the influenza virus infections during that time period were due to the 2009 H1 and H3 viruses. Hence, the evaluation of the influenza virus A H1 target was performed entirely by using contrived NPS samples. Similar to the BioFire RP results, instances of samples positive for influenza A virus without subtypes or positive for an influenza A virus subtype only were identified. Such results may be caused by low virus titers or a novel influenza virus subtype and thus require additional testing.

Only a limited number of samples were positive for Chlamydia pneumoniae, and the evaluation was conducted primarily with contrived samples. The performance for C. pneumoniae was similar to that reported in the BioFire RP clinical study, which was also conducted by using primarily contrived samples (12). Both Chlamydia pneumoniae and Mycoplasma pneumoniae are associated with atypical, community-acquired pneumonia (CAP), with no seasonality and nonspecific symptoms (18). Therefore, the inclusion of both bacteria in large multiplex panels has the potential to significantly improve the diagnosis of CAP.

A notable difference between the ePlex RP panel and the BioFire RP is the absence of Bordetella pertussis-Bordetella parapertussis targets among the bacterial targets. It is noteworthy that unlike other bacterial targets on the panel, infection with Bordetella pertussis-B. parapertussis has a relatively specific epidemiology, with higher incidences typically being reported for children less than 2 months old and, more recently, for unvaccinated populations or vaccinated patients with waning immunity (19). Thus, exclusion from a highly multiplexed respiratory panel may be warranted. In addition, the utilization of the single-copy pertussis toxin promoter target (ptxP) in some multiplex panels has been shown to be less sensitive for the detection of B. pertussis than PCR tests targeted to the multicopy IS*481* insertion sequence (20). As a result, when B. pertussis infection is suspected, an FDA-cleared B. pertussis molecular test may be more appropriate.

Similar to other broadly multiplexed respiratory panels, the ePlex RP panel allows the detection of coinfections. In this study, coinfections were identified in 5.5% of samples, with human rhinovirus/enterovirus being the most common virus detected in coinfected specimens. The importance of detecting coinfections was highlighted in a recent study showing that viral-bacterial coinfection during severe CAP in adults resulted in a more complicated disease course and was associated with complications (21). In other studies, human rhinoviruses were frequently detected in association with bacterial pathogens in a hematopoietic stem cell transplant population, suggesting that this commonly detected virus may be a significant cause of severe pneumonia in immunocompromised patients (22, 23).

The initial rate of invalid results with ePlex RP was 4.8%, with repeat testing resulting in a final rate of invalid results of 0.2%. This value falls in the range of rates of invalid results reported for other multiplexed respiratory panels, including Nanosphere Verigene RV (9.7%), xTAG RVP (1 to 2%), and the BioFire RP (1 to 3%) (24–27).

The ePlex instrument consists of a stand-alone system, with no additional accessories (e.g., computer) and a touch screen, making it a great option for space-constrained laboratories. The ePlex instrument is available in configurations ranging from one to four towers, with each tower containing six random-access testing bays. This supports single-shift (8 h) testing volumes ranging from 24 samples to 96 samples, with a TAT of less than 2 h and an HOT of less than 2 min, providing a flexible capacity for laboratories of all sizes. Additional features available on the system include the ability to generate customized reports (e.g., statistical and trending reports) and the potential for a bidirectional interface with laboratory information systems.

In conclusion, the ePlex RP panel provides a rapid, sensitive, broadly multiplexed, sample-to-results option with minimal hands-on time. The assay performance was equivalent to that of the BioFire FilmArray RP for all targets. This new, fully integrated instrument is easily expandable and offers a great option for the implementation of a multiplex assay in larger-volume laboratories.

## Supplementary Material

Supplemental material
