# Supplementary material for: Multicenter Evaluation of the ePlex Respiratory Pathogen Panel for the Detection of Viral and Bacterial Respiratory Tract Pathogens in Nasopharyngeal Swabs
Source: J Clin Microbiol. 2018 Jan 24;56(2):e01658-17. doi: 10.1128/JCM.01658-17 (PMC5786739; doi:10.1128/JCM.01658-17)
Supplement: Supplemental material [file supp_56_2_e01658-17__index.html]

Supplemental material 

# Multicenter Evaluation of the ePlex Respiratory Pathogen Panel for the Detection of Viral and Bacterial Respiratory Tract Pathogens in Nasopharyngeal Swabs

## Supplemental material

- Supplemental file 1 -

  Tables S1 (Prevalence of ePlex RP panel targets by age group during prospective collection with samples tested fresh, September to October 2016), S2 (Prevalence of ePlex RP panel targets by age group during prospective collection with samples tested after being frozen, March 2013 to August 2014), and S3 (Positive and negative percent agreement of the ePlex RP panel with comparator methods by organism)

  PDF, 100K
