# Supplementary material for: Multicenter Evaluation of the ePlex Respiratory Pathogen Panel for the Detection of Viral and Bacterial Respiratory Tract Pathogens in Nasopharyngeal Swabs
Source: J Clin Microbiol. 2018 Jan 24;56(2):e01658-17. doi: 10.1128/JCM.01658-17 (PMC5786739; doi:10.1128/JCM.01658-17)
Supplement: Supplemental material [file JCM.01658-17_zjm999095822s1.pdf]

SUPPLEMENTAL TABLES

Table S1. Prevalence of ePlex RP Panel Targets by Age Group During Prospective Collection With Samples Tested Fresh: September – October 2016

Organism	All Ages (N=511)	Age 0-1 (N=73)	Age >1-5 (N=75)	Age >5-21 (N=75)	Age >21-65 (N=181)	Age >65 (N=107)
Adenovirus	10 (2.0)	3 (4.1)	4 (5.3)	1 (1.3)	1 (0.6)	1 (0.9)
Coronavirus	8 (1.6)	2 (2.7)	0 (0.0)	1 (1.3)	4 (2.2)	1 (0.9)
Human Metapneumovirus	0 (0.0)	0 (0.0)	0 (0.0)	0 (0.0)	0 (0.0)	0 (0.0)
Human Rhinovirus/Enterovirus	188 (36.8)	37 (50.7)	40 (53.3)	33 (44.0)	58 (32.0)	20 (18.7)
Influenza A	0 (0.0)	0 (0.0)	0 (0.0)	0 (0.0)	0 (0.0)	0 (0.0)
Influenza A 2009 H1N1	0 (0.0)	0 (0.0)	0 (0.0)	0 (0.0)	0 (0.0)	0 (0.0)
Influenza A H1	0 (0.0)	0 (0.0)	0 (0.0)	0 (0.0)	0 (0.0)	0 (0.0)
Influenza A H3	0 (0.0)	0 (0.0)	0 (0.0)	0 (0.0)	0 (0.0)	0 (0.0)
Influenza B	2 (0.4)	0 (0.0)	0 (0.0)	1 (1.3)	1 (0.6)	0 (0.0)
Parainfluenza Virus 1	1 (0.2)	0 (0.0)	1 (1.3)	0 (0.0)	0 (0.0)	0 (0.0)
Parainfluenza Virus 2	13 (2.5)	3 (4.1)	4 (5.3)	3 (4.0)	2 (1.1)	1 (0.9)
Parainfluenza Virus 3	5 (1.0)	2 (2.7)	1 (1.3)	1 (1.3)	1 (0.6)	0 (0.0)
Parainfluenza Virus 4	8 (1.6)	1 (1.4)	4 (5.3)	2 (2.7)	1 (0.6)	0 (0.0)
Respiratory Syncytial Virus A	8 (1.6)	5 (6.8)	3 (4.0)	0 (0.0)	0 (0.0)	0 (0.0)
Respiratory Syncytial Virus B	9 (1.8)	3 (4.1)	4 (5.3)	0 (0.0)	2 (1.1)	0 (0.0)
Chlamydia pneumoniae	0 (0.0)	0 (0.0)	0 (0.0)	0 (0.0)	0 (0.0)	0 (0.0)
Mycoplasma pneumoniae	4 (0.8)	0 (0.0)	1 (1.3)	2 (2.7)	1 (0.6)	0 (0.0)

Table S2. Prevalence of ePlex RP Panel Targets by Age Group During Prospective Collection With Samples Tested After Being Frozen: March 2013 - August 2014

Organism	All Ages (N=1951)	Age 0-1 (N=315)	Age >1-5 (N=250)	Age >5-21 (N=246)	Age >21-65 (N=745)	Age >65 (N=395)
Adenovirus	72 (3.7)	31 (9.8)	24 (9.6)	7 (2.8)	7 (0.9)	3 (0.8)
Coronavirus	102 (5.2)	19 (6.0)	18 (7.2)	16 (6.5)	32 (4.3)	17 (4.3)
Human Metapneumovirus	113 (5.8)	22 (7.0)	28 (11.2)	6 (2.4)	31 (4.2)	26 (6.6)
Human Rhinovirus/Enterovirus	388 (19.9)	113 (35.9)	94 (37.6)	58 (23.6)	87 (11.7)	36 (9.1)
Influenza A	110 (5.6)	6 (1.9)	18 (7.2)	20 (8.1)	49 (6.6)	17 (4.3)
Influenza A 2009 H1N1	76 (3.9)	4 (1.3)	13 (5.2)	14 (5.7)	37 (5.0)	8 (2.0)
Influenza A H1	0 (0.0)	0 (0.0)	0 (0.0)	0 (0.0)	0 (0.0)	0 (0.0)
Influenza A H3	34 (1.7)	1 (0.3)	5 (2.0)	6 (2.4)	12 (1.6)	10 (2.5)
Influenza B	62 (3.2)	4 (1.3)	9 (3.6)	10 (4.1)	24 (3.2)	15 (3.8)
Parainfluenza Virus 1	24 (1.2)	4 (1.3)	12 (4.8)	4 (1.6)	3 (0.4)	1 (0.3)
Parainfluenza Virus 2	10 (0.5)	4 (1.3)	4 (1.6)	0 (0.0)	2 (0.3)	0 (0.0)
Parainfluenza Virus 3	99 (5.1)	31 (9.8)	20 (8.0)	3 (1.2)	27 (3.6)	18 (4.6)
Parainfluenza Virus 4	7 (0.4)	3 (1.0)	2 (0.8)	1 (0.4)	1 (0.1)	0 (0.0)
Respiratory Syncytial Virus A	28 (1.4)	13 (4.1)	6 (2.4)	3 (1.2)	2 (0.3)	4 (1.0)
Respiratory Syncytial Virus B	83 (4.3)	33 (10.5)	19 (7.6)	6 (2.4)	15 (2.0)	10 (2.5)
Chlamydia pneumoniae	3 (0.2)	0 (0.0)	0 (0.0)	1 (0.4)	1 (0.1)	1 (0.3)
Mycoplasma pneumoniae	5 (0.3)	1 (0.3)	1 (0.4)	2 (0.8)	1 (0.1)	0 (0.0)

Table S3. Positive and Negative Percent Agreement of the ePlex RP Panel With Comparator Methods by Organism

Adenovirus		Positive % Agreement TP/TP+FN	% (95% CI)	Negative % Agreement TN/TN+FP	NPA (95% CI)
Prospectively-Collected Samples	Fresh	6/8[a]	75.0 (40.9-92.9)	499/503[a]	99.2 (98.0-99.7)
	Frozen	48/53[b]	90.6 (79.7-95.9)	1874/1898[b]	98.7 (98.1-99.1)
	TOTAL	54/61	88.5 (78.2-94.3)	2373/2401	98.8 (98.3-99.2)
**Coronavirus**					
Prospectively-Collected Samples	Fresh	7/7	100 (64.6-100)	503/504	99.8 (98.9-100)
	Frozen	89/110[c]	80.9 (72.6-87.2)	1828/1841[d]	99.3 (98.8-99.6)
	TOTAL	96/117	82.1 (74.1-88.0)	2331/2345	99.4 (99.0-99.6)
**Human Metapneumovirus**					
Prospectively-Collected Samples	Fresh	0/0	---	511/511	100 (99.3-100)
	Frozen	107/113[e]	94.7 (88.9-97.5)	1832/1838[e]	99.7 (99.3-99.9)
	TOTAL	107/113	94.7 (88.9-97.5)	2343/2349	99.7 (99.4-99.9)
**Human Rhinovirus/Enterovirus**					
Prospectively-Collected Samples	Fresh	176/183[f]	96.2 (92.3-98.1)	316/328[f]	96.3 (93.7-97.9)
	Frozen	317/336[g]	94.3 (91.3-96.4)	1544/1615[g]	95.6 (94.5-96.5)
	TOTAL	493/519	95.0 (92.8-96.6)	1860/1943	95.7 (94.7-96.5)
**Influenza A**					
Prospectively-Collected Samples [h]	Fresh	0/0	---	511/511	100 (99.3-100)
	Frozen	106/111[i]	95.5 (89.9-98.1)	1836/1840[i]	99.8 (99.4-99.9)
	TOTAL	106/111	95.5 (89.9-98.1)	2347/2351	99.8 (99.6-99.9)
**Influenza A H1**					
Prospectively-Collected Samples	Fresh	0/0	---	511/511	100 (99.3-100)
	Frozen	0/0	---	1951/1951	100 (99.8-100)
	TOTAL	0/0	---	2462/2462	100 (99.8-100)
**Influenza A 2009 H1N1**					
Prospectively-Collected Samples	Fresh	0/0	---	511/511	100 (99.3-100)
	Frozen	70/71	98.6 (92.4-99.8)	1874/1880[j]	99.7 (99.3-99.9)
	TOTAL	70/71	98.6 (92.4-99.8)	2385/2391	99.7 (99.5-99.9)
**Influenza A H3**					
Prospectively-Collected Samples	Fresh	0/0	---	511/511	100 (99.3-100)
	Frozen	34/37[k]	91.9 (78.7-97.2)	1914/1914	100 (99.8-100)
	TOTAL	34/37	91.9 (78.7-97.2)	2425/2425	100 (99.8-100)
**Influenza B**					
Prospectively-Collected Samples	Fresh	1/1	100 (20.7-100)	509/510	99.8 (98.9-100)
	Frozen	58/65[l]	89.2 (79.4-94.7)	1882/1886[l]	99.8 (99.5-99.9)
	TOTAL	59/66	89.4 (79.7-94.8)	2391/2396	99.8 (99.5-99.9)

			**PPA**	**Negative % Agreement**	
**Parainfluenza Virus 1**		**TP/TP+FN**	**% (95% CI)**	**TN/TN+FP**	**NPA (95% CI)**
Prospectively-Collected Samples	Fresh	1/1	100 (20.7-100)	510/510	100 (99.3-100)
	Frozen	23/24	95.8 (79.8-99.3)	1926/1927	99.9 (99.7-100)
	TOTAL	24/25	96.0 (80.5-99.3)	2436/2437	100 (99.8-100)
**Parainfluenza Virus 2**					
Prospectively-Collected Samples	Fresh	12/13	92.3 (66.7-98.6)	497/498	99.8 (98.9-100)
	Frozen	9/9	100 (70.1-100)	1941/1942	99.9 (99.7-100)
	TOTAL	21/22	95.5 (78.2-99.2)	2438/2440	99.9 (99.7-100)
**Parainfluenza Virus 3**					
Prospectively-Collected Samples	Fresh	5/5	100 (56.6-100)	506/506	100 (99.2-100)
	Frozen	94/104[m]	90.4 (83.2-94.7)	1842/1847[m]	99.7 (99.4-99.9)
	TOTAL	99/109	90.8 (83.9-94.9)	2348/2353	99.8 (99.5-99.9)
**Parainfluenza Virus 4**					
Prospectively-Collected Samples	Fresh	3/3	100 (43.9-100)	503/508[n]	99.0 (97.7-99.6)
	Frozen	5/5	100 (56.6-100)	1944/1946	99.9 (99.6-100)
	TOTAL	8/8	100 (67.6-100)	2447/2454	99.7 (99.4-99.9)
**Respiratory Syncytial Virus A**					
Prospectively-Collected Samples	Fresh	8/9	88.9 (56.5-98.0)	501/501	100 (99.2-100)
	Frozen	27/31	87.1 (71.1-94.9)	1917/1918	99.9 (99.7-100)
	TOTAL	35/40	87.5 (73.9-94.5)	2418/2419	100 (99.8-100)
**Respiratory Syncytial Virus B**					
Prospectively-Collected Samples	Fresh	9/10	90.0 (59.6-98.2)	500/500	100 (99.2-100)
	Frozen	81/86	94.2 (87.1-97.5)	1861/1863[o]	99.9 (99.6-100)
	TOTAL	90/96	93.8 (87.0-97.1)	2361/2363	99.9 (99.7-100)
Chlamydia pneumoniae					
Prospectively-Collected Samples	Fresh	0/0	---	511/511	100 (99.3-100)
	Frozen	2/5[p]	40.0 (11.8-76.9)	1945/1946[p]	99.9 (99.7-100)
	TOTAL	2/5	40.0 (11.8-76.9)	2456/2457	100 (99.8-100)
Mycoplasma pneumoniae					
Prospectively-Collected Samples	Fresh	3/3	100 (43.9-100)	507/508[q]	99.8 (98.9-100)
	Frozen	4/5[r]	80.0 (37.6-96.4)	1945/1946	99.9 (99.7-100)
	TOTAL	7/8	87.5 (52.9-97.8)	2452/2454	99.9 (99.7-100)

[a] Adenovirus was not detected in 2 of 2 FN samples and detected in 4 of 4 FP samples using PCR/sequencing.
[b] Adenovirus was not detected in 1 of 5 FN samples and detected in 9 of 24 FP samples using PCR/sequencing.
[c] Coronavirus was not detected in 2 of 21 FN samples and detected in 3 of 13 FP samples using PCR/sequencing.
[e] Human metapneumovirus was not detected in 1 of 6 FN samples and detected in 4 of 6 FP samples using PCR/sequencing.
[f] Human rhinovirus/enterovirus was not detected in 1 of 7 FN samples and detected in 9 of 12 FP samples using PCR/sequencing.
[g] Human rhinovirus/enterovirus was not detected in 6 of 19 FN samples and detected in 33 of 71 FP samples using PCR/sequencing.

[h]Influenza A comparator results contain 71 samples with A 2009 H1N1, 37 samples with A H3, and 3 samples with no subtype detected
[i] Influenza A was not detected in 1 of 3 FN samples (2 samples were not tested) and detected in 1 of 4 FP samples using PCR/sequencing.
[j] Influenza A H1-2009 was detected in 4 of 6 FP samples using PCR/sequencing
[k] Influenza A H3 was not detected in 1 of 3 FN samples using PCR/sequencing.
[l] Influenza B was not detected in 3 of 7 FN samples and detected in 2 of 4 FP samples using PCR/sequencing.
[m] Parainfluenza virus 3 was not detected in 3 of 10 FN samples and detected in 4 of 5 FP samples using PCR/sequencing
[n] Parainfluenza virus 4 was detected in 3 of 5 FP samples using PCR/sequencing.
[o]RSV B was detected in 1 of 2 FP samples using PCR/sequencing.
[p] *C. pneumoniae* was not detected in 1 of 3 FN samples and detected in the 1 FP sample using PCR/sequencing.
[q] *M. pneumoniae* was detected in the 1 FP sample using PCR/sequencing.
[r] *M. pneumoniae* was not detected in the 1 FN sample using PCR/sequencing.
